# Examining problem gambling, substance use disorders and cluster B personality traits among incarcerated individuals

**DOI:** 10.1016/j.abrep.2024.100566

**Published:** 2024-10-09

**Authors:** Kalle Lind, Jussi Palomäki, Sari Castrén

**Affiliations:** a Finnish Institute for Health and Welfare, Department of Public Health and Welfare, Helsinki, Finland; b Cognitive Science, Department of Digital Humanities, Faculty of Arts, University of Helsinki, Helsinki, Finland; c Social Sciences Department of Psychology and Speech-Language Pathology Turku, University of Turku, Turku, Finland; d Department of Medicine, University of Helsinki, Helsinki, Finland

**Keywords:** Criminal behavior, Incarcerated persons, Prison population, Problem gambling, Risk

## Abstract

Prison populations face heightened rates of addictive behaviors and significant stressors, including health challenges and social disparities. Although problem gambling is prevalent in criminal justice settings, its distinctiveness compared to other addictions among incarcerated individuals remains uncertain. This study examines risk factors associated with problem gambling in Finnish prisoners and explores differences compared to other forms of addiction.

Covariates from a questionnaire (N = 527) included gender, age, education, offender status, involvement in theft or property crime, Trauma Screening Questionnaire (TSQ), Depression Scale (DEPS), and lifetime abuse experiences. Of the 527 participants, 330 were interviewed using the Structured Clinical Interview (SCID-II) to assess personality disorders. Multiple logistic regression models were utilized, with separate models for problem gambling (PG), lifetime substance use disorder, and lifetime alcohol disorder.

Crime type was not significantly associated with alcohol or substance use problems, but PG was associated with property crime or theft convictions. Borderline personality traits were linked to probable PG, while antisocial traits were linked to drug use, and both traits predicted alcohol misuse. Age and gender were significant only in relation to alcohol use problems, with older age and male gender indicating higher risk.

The association between problem gambling and criminal behavior underscores the vulnerability of incarcerated individuals across various domains. Detailed assessments, considering the nature of criminal activity and personality disorders, especially borderline traits and comorbid conditions, are crucial for tailoring rehabilitation and treatment strategies to prevent recidivism. These findings provide valuable insights for clinicians managing addiction and mental health issues within correctional settings.

## Highlights

• A conviction for theft or property crime predicted probable problem gambling.
• Borderline traits predict problem gambling.
• Antisocial traits predicted lifetime drug use problems, and both predict lifetime alcohol problems.
• Understanding these factors helps tailor interventions for incarcerated individuals, reducing recidivism.

## 1 Introduction

Problem gambling (PG) is remarkably prevalent among individuals under criminal sanctions and is significantly associated with recidivism. Furthermore, prisoners frequently exhibit other addictive behaviors, such as substance use disorders (SUDs), along with various psychiatric comorbidities. Therefore, it is important to better understand the associations between addictive behaviors and psychiatric comorbidities to develop effective preventive strategies and to mitigate the risk of recidivism.

### 1.1 Gambling in Finland

According to the latest population survey by the Finnish Institute for Health and Welfare (THL, 2024), approximately 70 % of adults in Finland have engaged in gambling over the past 12 months. Among the different game types, lotteries, slot machines, sports betting, and online casino games have remained popular in Finland, lotteries and slot machine gambling being especially prevalent, influenced by their extensive availability and accessibility. Online gambling is becoming increasingly popular. Population prevalence of moderate risk or problem gambling (scoring 3 or more points on the Problem Gambling Severity Index) was 4.2 % in the 2024 survey. The prevalence of low-risk gambling, defined as scoring 1–2 points on the PGSI, has seen a significant increase, rising from 6.7 % in 2019 to 9.0 % in 2023.

While gambling is a common pastime across the population, gambling expenditure is disproportionately cumulated to individuals who gamble at moderate-risk or problem levels. Socially disadvantaged individuals are overrepresented in this group (Grönroos et al., 2021, Latvala et al., 2021).

### 1.2 Problem gambling and substance use among incarcerated individuals

The prevalence of PG among incarcerated persons has previously ranged from 5.9 % to 73 % and is significantly higher compared to the general population (Banks et al., 2020; Adolphe et al., 2019). This can be compared to the pooled prevalence estimate of 30.8 % in the *meta*-analysis by Tørdal et al. (2024). Similarly, individuals with a history of drug use are overrepresented in prison settings (Montanari et al., 2014), with around half of European inmates reporting drug use in the year leading to incarceration (Favril, 2023; van de Baan et al., 2022) and a substantial portion, ranging from 30 % to 51 %, meeting the diagnostic criteria for a drug use disorder upon entry into prison (Fazel et al., 2017).

The prevalence estimates for PG and substance use are higher among incarcerated males than females reported in most of the studies (Lind et al., 2019; Banks et al., 2020, Dalbir et al., 2022, Fazel et al., 2017) yet the opposite gender pattern was found in a Norwegian sample regarding drug use (Bukten et al., 2020).

Individuals incarcerated with PG and Substance Use Disorders (SUDs) often face a myriad of mental and social challenges, such as decreased educational attainment, lower level of employment, housing instability, compromised physical well-being, as well as increased behavioral, psychological, and psychiatric issues (Riley & Oakes, 2015; Dolan et al., 2018).

### 1.3 Mental health, psychiatric comorbidities, personality traits and traumatic life experiences

The co-occurrence of comorbid mental illnesses with PG and SUDs is intricately tied to adverse outcomes across criminal, social, and health domains among the prison population (Baranyi et al., 2022, Brown et al., 2022; Lind et al., 2019).

Individuals with personality disorders (PDs) are susceptible to PG. PDs are a group of severe mental disorders characterized by lasting dysfunctional patterns of cognition, affect regulation, interpersonal functioning, and impulse control. These patterns are pervasive across a broad range of personal and social situations and cause considerable personal distress based on the Diagnostic and Statistical Manual of Mental Disorders (5th ed.; DSM–5; American Psychiatric Association, [APA] 2013). The DSM-5-TR delineates ten distinct personality disorders. These disorders represent enduring patterns of inner experience and behavior that deviate markedly from the normative expectations of an individual's culture. Personality disorders are comprised of three clusters: Cluster A includes paranoid, schizoid and schizotypal disorders; Cluster B includes antisocial personality disorder (ASPD), borderline personality disorder (BPD), histrionic and narcissistic disorders and Cluster C includes avoidant, dependent and obsessive–compulsive personality disorders (APA, 2013).

Antisocial Personality Disorder (ASPD) is defined by persistent behavior patterns exhibiting disregard for and violation of others' rights, deceitfulness, manipulation, and impulsivity that begin by age 15, coupled with a history of conduct disorder prior to age 15. Conversely, BPD is understood as a disorder characterized by emotional dysregulation, impulsivity, suicidality, identity disturbance, and significant interpersonal relationship difficulties (APA, 2013).

Evidence suggests that Cluster B personality disorders (BPD, ASPD), as well as SUDs, are relatively prevalent among incarcerated individuals (Wetterborg et al., 2015, Armenti et al., 2017) and are linked to PG (Blaszczynski and Nower, 2002, Pastwa-Wojciechowska, 2011). Numerous studies have suggested that many factors like co-morbid mental disorders could mediate the connection between gambling and criminal offences (Preston et al., 2012, Meyer & Stadler, 1999). Gorsane et al. (2016) suggest that concurrent substance use alongside PG could reduce inhibitions, potentially resulting in unlawful behavior.

Compared to other incarcerated individuals, those with co-occuring mental disorders and SUDs (‘dual diagnosis’) exhibit more severe criminal backgrounds, often marked by prior incarcerations (Borschmann et al., 2020) and violent offenses (Wilton & Stewart, 2017), as well as elevated rates of significant institutional infractions while imprisoned (Wilton & Stewart, 2017). The prevalence of dual diagnosis is higher among women than men (Borschmann et al. 2020), and they are more frequently found among survivors of childhood physical and sexual abuse (Villagra et al., 2019) who intersect with the criminal justice system. These disorders correlate with various physical health issues (Borschmann et al., 2020) and impairments in psychosocial, cognitive, and occupational functioning (Substance Abuse and Mental Health Services Administration, 2015). Post-release, individuals with dual diagnosis face a considerably increased risk of reincarceration (Wilton and Stewart, 2017, Baillargeon et al., 2010), suicide attempts (Gates et al., 2017), and hospitalization due to injuries, compared to their counterparts without such disorders (Young et al., 2018). Dual diagnosis present significant challenges for treatment planning, and frequently, criminal justice systems lack the infrastructure and resources required to effectively manage these complexities.

Certain personality characteristics and traumatic life events (Young et al., 2018, Degenhardt et al., 2022) may interact to enhance an individual's vulnerability to a range of risk factors. Traumatic life events such as negative adverse childhood experiences and problems with emotion regulation are common among incarcerated individuals and predispose them to the development of gambling disorders (Estevez et al., 2023; Richard et al., 2023; Afifi et al., 2010; Boughton & Falenchuk, 2007). Childhood physical neglect (Horak et al., 2021, Hodgins et al., 2010) is associated with PG and gambling disorder (Scherrer et al., 2007). Experiencing traumatic events can lead some individuals to seek out gambling to cope with their emotional pain and distress. In fact, lack of emotional awareness maintains various other addictions, but this effect is strongest for PG (e.g., Panayiotou et al., 2023). Individuals who have difficulties with emotion regulation and gambling-related erroneous beliefs are highly vulnerable to gambling problems when they face traumatic or stressful life events (Thurm et al., 2022).

### 1.4 Crime type related to problem gambling

Some studies suggest that incarcerated individuals with PG-related crimes are distinct from other incarcerated persons. In a study of Swedish court documents of gambling-related embezzlements (Binde et al., 2022), older individuals, females and first timers were overrepresented compared to overall crime statistics in Sweden. PG and criminal behavior can either have a direct relationship due to financial difficulties (PG leading to the sentenced crime, e.g., embezzled money to pay off a gambling debt or to accumulate funds to gamble) (Ledgerwood & Petry, 2006) or they might simply co-exist as different displays of risk-taking. Generally, crimes committed by persons with PG are non-violent and driven by the need to obtain gambling funds (Turner et al., 2009; Adolphe et al., 2019). Additionally, among the general population, criminal convictions appear to be more prevalent among those who meet the criteria for PG (Lind et al., 2024).

A relevant theoretical perspective in this realm is The Pathways Model, proposed by Blaszcynski and Nower in 2002, which is a comprehensive framework that classifies problem gamblers into three distinct subtypes based on the interplay of psychological, genetic, and environmental factors. Each subtype represents a unique pathway leading to the development of problem gambling behavior. This model offers valuable insights for understanding and addressing problem gambling in various settings, including prisons.

The three subtypes are: 1) behaviorally conditioned gamblers, 2) emotionally vulnerable gamblers and 3) antisocial impulsive gamblers. The most relevant subgroups among incarcerated individuals are groups 2 and 3.

In subgroup 2 individuals gamble as a means of coping with emotional distress, psychological disorders, or adverse life experiences. They may have underlying issues such as depression, anxiety, trauma, or a history of abuse that drive them towards gambling as an escape mechanism. Risk factors include pre-existing psychological conditions, traumatic life events, poor coping strategies, and comorbid mental health disorders. Given the high prevalence of emotional and psychological distress among incarcerated individuals, some may use gambling to cope with the harsh realities of prison life, unresolved trauma, or mental health issues. This subtype may be particularly prevalent among inmates who have experienced childhood abuse or other significant adverse experiences.

In subgroup 3 individuals exhibit high levels of impulsivity and antisocial behaviors, which contribute to their gambling problems. They are often characterized by a lack of self-control, a propensity for risk-taking, and comorbid personality disorders. Risk factors include antisocial personality traits, impulsivity, substance use disorders, and a predisposition towards criminal behavior. Incarcerated individuals with antisocial impulsive traits may be more likely to engage in gambling as part of a broader pattern of impulsive and risky behaviors. The structured prison environment might temporarily mitigate some external opportunities for risk-taking but could also intensify internal drives that contribute to gambling problems.

In this paper, we focus on individuals with probable PG, defined by the Brief Biosocial Gambling Screen (BBGS; Gebauer et al., 2010) addressing the distinct vulnerability factors based on the Pathways model (Blaszcynski & Nower, 2002). Our objective is to discover the distinct correlates associated with probable PG and lifetime substance use disorders (SUD), exploring variations in age, gender, education, primary crime type, first-offense status, experiences of trauma, lifetime abuse, work capacity, and mental health problems such as depression, personality disorders (BPD and ASPD traits). Our findings carry substantial implications for informing the design of tailored support within the context of criminal sanctions. We hope that our findings will contribute to developing efficient measures for risk assessment and early screening, tailored interventions and preventive measures, and support in a prison setting.

## 2 Method

This study utilizes data from the Health and Wellbeing of Finnish Prisoners 2023 study coordinated by the Finnish Institute for Health and Welfare. The data was collected from the convict prisoner population between 2020 and 2022. In the Finnish criminal justice system different forms of punishments include fines, community service, probation, and both conditional and unconditional imprisonment. In addition, prison populations consist of remand prisoners, who are imprisoned in prison due to a suspected offence, and those with conversion sentences when a fine cannot be collected as a sum of money. Unconditional imprisonment can be sentenced as a fixed term or life imprisonment. The convict prisoner population consists of those individuals carrying out their unconditional fixed-term or life prison sentences. Our data includes only convict prisoners due to other sentences being too short or unpredictable for data collection. For a clearer insight into the data collection procedures, consult the “Health and Wellbeing of Finnish Prisoners 2023” report for detailed information (Rautanen et al., 2024). The study included both closed prisons and open prisons. The study aimed for a representative sample by including various sentenced prisoners, prison types and geography. COVID-19 restrictions skewed the sample towards long-term prisoners and limited geographic reach. Despite these issues, the sample remained consistent with broader Finnish prisoner statistics from the Prison and Probation Services, making the findings largely generalizable to the prisoner population post-COVID-19.

The data collection contained different modules. The survey included various items about the respondents’ demographic background, health, and psychosocial situation. The variables chosen for this study consist mainly of survey items. In addition to the health survey, the study included various physical examinations, ranging from blood tests to dental examinations and clinical interviews. Psychiatric nurses and psychologists conducted the mental health interviews for those participants, who agreed to be interviewed. In this study, in addition to the survey items, we also use information about the respondents’ substance use and personality disorders evaluated in clinical interviews.

The study was conducted in accordance with the ethical standards of the Declaration of Helsinki. The Ethics Committee of the Finnish Institute for Health and Welfare, Finland, approved the research protocol.

## 3 Measures

### 3.1 Dependent variables

There were three dichotomous dependent variables (DVs): dichotomized scores on the Brief Biosocial Gambling Screen (BBGS, Gebauer et al., 2010; scores = 0 were coded as “0″ [N = 455], and scores > 0 as “1” [N = 65]), a lifetime drug use problem (N_no_ = 77, N_yes_ = 218), a lifetime alcohol use problem (N_no_ = 93, N_yes_ = 202). Note that the BBGS was incorporated into the questionnaire, while lifetime substance use disorders were assessed through clinical interviews, in which not all survey respondents took part.

The Brief Biosocial Gambling Screen (BBGS, Gebauer et al., 2010) was included in The Health and Wellbeing of Finnish Prisoners 2023 survey questionnaire (n = 527) for the first time in the survey’s history. Using three items (withdrawal, deception, and bailout), the BBGS evaluates probable PG during the past year. Cronbach's alpha for the scale was 0.771. Although gambling is common during prison time, legal gambling is restricted or non-existent in prisons. Thus, the timeframe was modified to “12 months before the implementation of your sentence” instead of the past 12 months (as in our previous pilot study, Lind et al. 2020). The first item was modified to inquire whether the respondent had gambled in the first place. The BBGS items in the questionnaire were:

• **Withdrawal, neuroadaptation:** “During the 12 months before the implementation of your sentence, did you become restless, irritable or anxious when trying to stop/cut down on gambling?” (Yes/No/I do not gamble)
• **Deception, psychosocial characteristics:** “During the 12 months before the implementation of your sentence, did have you try to keep your family or friends from knowing how much you gambled?” (Yes/No)
• **Bailout, adverse social consequences of gambling:** “During the 12 months before the implementation of your sentence, did you have such financial trouble as a result of your gambling that you had to get help with living expenses from family, friends or welfare?” (Yes/No)

Each positive answer scores a point. The overall score thus varies from zero to three. One point or more suggests probable PG. Whether their current crime was gambling related, however, is not known.

### 3.2 Covariates

The survey included various items about the subjects’ background, health, and psychosocial situation (see Appendix 1∙for a full list). The Depression Scale (DEPS) was employed for evaluating depressive symptoms, consisting of a 10-item questionnaire with four response options (0–3: Not at all/A little/Quite a lot/Extremely) for each item. Unfortunately, due to a questionnaire error, one item (“I have had feelings of worthlessness”) was omitted from the form. Consequently, validated cut-offs were not applicable, and the scale was utilized as a continuous variable. The scale has demonstrated good sensitivity and specificity in assessing clinical depression, with varying performance in different patient populations. The scale achieved a Cronbach's alpha of 0.900, demonstrating very good internal consistency.

The 10-item Trauma Screening Questionnaire (TSQ) was used to assess experienced trauma. Individuals who had encountered traumatic events were queried as to whether the traumatic symptoms had recurred at least twice in the preceding week. Assessment was based on participants’ responses to each item, with a score of 1 for “yes” and 0 for “no.” We used 6 points or more as a cut-off: those scoring at least 6 points may be indicative (see Brewin et al., 2002) of trauma that could potentially result in post-traumatic stress disorder (dichotomous 6 points or more or 5 points or less). Cronbach's alpha for the 10-item scale was 0.864, indicating very good internal consistency.

The questionnaire included items pertaining to various forms of abuse, namely mental, physical, and sexual abuse. Respondents were asked to indicate the occurrence of these experiences for different life phases, with response options categorized as follows: “no,” “yes, under 18 years of age,” “yes, as an adult over 18 years of age,” “yes, both as a child and adult,” and “yes, in prison.” The responses were then coded as dichotomous variables to reflect lifetime experiences of different forms of abuse (yes/no).

Personality disorders were assessed using the Structured Clinical Interview for DSM-IV Axis II Personality Disorders (SCID-II). This is a tool used in clinical settings for the assessment of personality disorders. It is part of the SCID series, which stands for Structured Clinical Interview for DSM (Diagnostic and Statistical Manual of Mental Disorders). In our models, we utilized counted frequencies of endorsed traits for BPD and ASPD. Of all the participants, 330 agreed to be interviewed.

We also included gender (male or female), age (dichotomous: above or below 30), education (dichotomous: at most basic schooling or above basic schooling), whether the subject was a first time or repeat offender (dichotomous), and whether the subject had committed theft or a property crime (dichotomous: has committed either or has not committed either) in the models.

### 3.3 Statistical analyses

All analyses were conducted using the R platform for statistical computing (v. 4.2.1, R Core Team). We used multiple logistic regression modelling, fitting separate models for each DV. Based on visual inspection, the logit of the DV had a linear association with all continuous variables of interest in all models. The generalized variance inflation factor (VIF) values ranged between 1.03 and 1.89, suggesting there were no issues of multicollinearity.

Out of the 527 survey respondents, 7 did not complete the BBGS. Among the survey respondents, 330 took part in the SCID-II interviews. Missing values are omitted listwise in the analyses. Thus, in our final analyses the sample size was either 327 (with dichotomous BBGS as the DV) and 288 (with lifetime use of drugs or alcohol as the DV). For effect size estimates we used Cragg-Uhler and McFadden pseudo-r^2^ −values.

## 4 Results

### 4.1 Descriptives

Of all the respondents, 49 % reported to have gambled. Gambling was more common among men (51 %), compared to women (42 %). The gambling prevalence was higher (54 %) among those respondents who had only a basic education background compared to those who had a higher education (43 %). Considering different main crime types, gambling was most common (63 %) among respondents who were convicted of theft or property crimes, compared to those who were convicted of other crimes (47 %).

Of all the respondents, 13 % (n = 65) were individuals with probable PG (PPGs, 26 % of respondents who had gambled) based on their BBGS score (1 or more). PPGs were younger on average (mean 33.9; SD 9.2) compared to non-PPGs (mean 37.5; SD 10.8). Over 90 % of PPGs were younger than 47 years. Of the respondents under 30 years old, 17 % were PPGs. For first-timers this figure was 14 %. Of those respondents with previous sentences, the prevalence of PPGs was 11 %. Of the respondents with theft or property crime as their main crime, 22 % were PPGs (drug-related offences 12 %, other main crime types 11 %).

### 4.2 Logistic regression analyses

Having committed theft or property crimes, lower TSQ scores, and a higher number of BPD personality indicators were associated with gambling problems (dichotomous with the BBGS-scale: 0 points vs. 1 point or more).

A higher number of ASPD indicators was associated with lifetime drug use. In this model, the effects of education, age, and first-timer status were diluted by including the ASPD variable. The model fit remains high, but some associations with demographic variables are obscured.

**Fig. 1:**
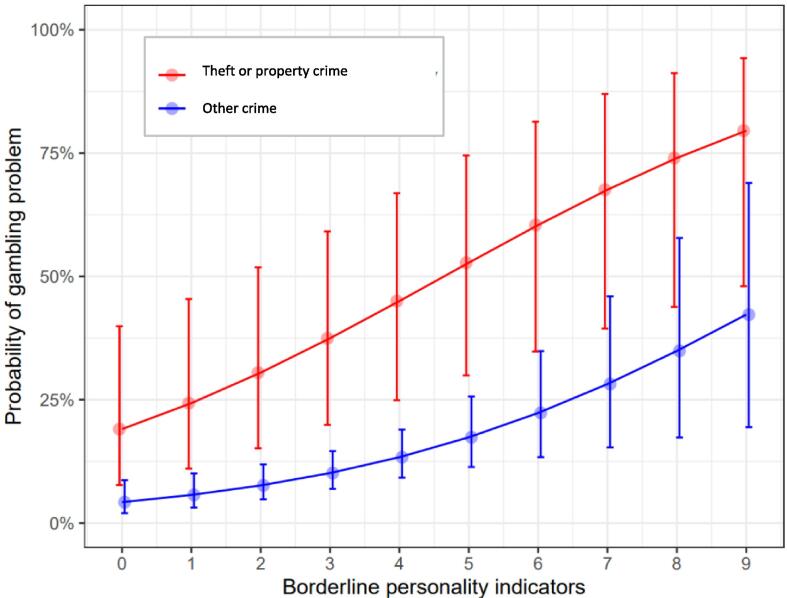
Association between borderline personality traits, crime type, and probability of PG (BBGS scores)*Note.* The error bars are 95% confidence intervals from a logistic regression model controlling for all other independent variables.

Male gender, older age, inability to work, and a higher number of both BPD and ASPD traits were associated with lifetime alcohol use problem. See Table 1 and Fig. 1 for full details of the analyses.

**Table 1:** Multiple logistic regression model with I) BBGS, II) lifetime drug use problem, and III) lifetime alcohol use problem as the dependent variables in separate models.

	**Odds ratio (95 % confidence interval)**		
**Dependent variable→**	**BBGS > 0**	**Lifetimedrug use problem**	**Lifetimealcohol use problem**
**Independent variable**			
*(Intercept)*	0.02 (0.00–0.34)*	0.54 (0.07–3.74)	0.08 (0.01–0.57)*
Gender (ref: Male)	0.46 (0.13–1.39)	1.04 (0.42–2.70)	0.23 (0.09–0.54)***
Age (ref: Under 30)	0.57 (0.27–1.23)	0.49 (0.2–1.11)	2.49 (1.25–5.03)**
Education (ref: at most basic schooling)	1.28 (0.62–2.66)	0.74 (0.38–1.45)	0.84 (0.46–1.54)
First timer (ref: Repeat offender)	1.03 (0.46–2.27)	0.55 (0.27–1.11)	1.88 (0.99–3.69)
Theft/property crime (ref: No)	5.3 (1.94–14.1)***	1.32 (0.38–5.49)	0.39 (0.13–1.13)
TSQ (Ref: 6 pts or more)	0.31 (0.11–0.82)*	1.63 (0.65–4.27)	0.89 (0.41–1.98)
Depression (DEPS) score	1.00 (0.93–1.08)	0.98 (0.91–1.05)	1.03 (0.97–1.10)
Mental abuse (ref: No)	0.87 (0.40–1.92)	0.62 (0.28–1.32)	0.76 (0.38–1.49)
Physical abuse (ref: No)	2.70 (0.46–52.9)	1.23 (0.33–4.72)	2.71 (0.77–10.06)
Sexual abuse (ref: No)	0.99 (0.34–2.7)	0.85 (0.34–2.12)	1.85 (0.80–4.54)
Ability to work (ref: No)	1.34 (0.64–2.85)	1.38 (0.71–2.72)	0.49 (0.26–0.91)*
Borderline personality	1.36 (1.13–1.65)**	1.19 (0.98–1.47)	1.22 (1.03–1.45)**
Antisocial personality	0.99 (0.77–1.28)	1.78 (1.41–2.28)***	1.37 (1.11–1.71)*

**Model fit**			
Cragg-Uhler pseudo R^2^	0.19	0.37	0.28
McFadden pseudo R^2^	0.13	0.25	0.18

*Note.* * p < 0.05, ** p < 0.01, *** p < 0.001. Statistically significant cells for predictors highlighted in shades of green, darker shades meaning more significant.

## 5 Discussion

To our knowledge, this is the first study to explore relationships between probable PG, distress, substance use problems and specifically Cluster B personality disorders (BPD and ASPD personality disorders) in a nationally representative prison population. We identified different factors associated with probable PG compared to lifetime drug use and alcohol use problems. Crime type did not significantly relate to substance or alcohol use problems, but individuals sentenced for property crime or theft were more likely to score on the Brief Biosocial Gambling Screen (BBGS). BPD traits were linked with probable PG, while antisocial personality disorder traits were associated with drug use problems, and both personality disorders were correlated with alcohol use problems. Age and gender were significant factors only in models where alcohol use problems were the outcome, with older age and male gender associated with greater issues with alcohol use.

These results align with prior research indicating a connection between PG, certain types of crime, and BPD traits (Turner et al., 2021, Brown et al., 2015) and additional findings, such as the entanglement of PDs and PG, that could be considered when designing rehabilitation programs for incarcerated individuals. Multiple factors play a role in leading an individual to incarceration and therefore it is very important to address the core of the problems thoroughly. The association we identified between property crime or theft and PG appears to be attributable, in substantial part, which can be explained by strong monetary motivation in PG yet is also apparent with SUDs related to the need to finance drug use. (Stewart et al., 2000, Sutherland et al., 2015). PG often goes unnoticed and underdiagnosed (Salonen et al., 2022; Karlsson & Håkansson, 2018; Håkansson et al., 2018), remaining elusive due to its intangible nature, and with no pathognomonic physical symptoms present, despite the multiple harms experienced (Langham et al., 2015).

Gambling behavior is frequently motivated by financial factors such as seeking more money to gamble with, trying to recoup losses, or resolving gambling-related debts (Ledgerwood & Petry, 2006; Clark et al., 2009; Mathieu et al., 2020, Roberts et al., 2021). Thus, it is crucial to identify potential gambling issues among incarcerated individuals through screening for PG and its connection to types of crime. In contrast to the recent study by Roberts et al. (2021), our investigation did not establish a correlation between a history of abuse, depressive symptoms, or lower educational attainment as risk factors for PG. Notably, Roberts et al. (2021) observed that individuals who had engaged in criminal behavior to fund their gambling demonstrated an association with PG. The disparity in these findings could potentially be attributed to variations in sample characteristics, with one study comprising treatment-seeking individuals and the other employing a more general questionnaire approach. Individuals who resort to criminal activities to fund their gambling behavior may possess distinct treatment requirements, necessitating tailored clinical interventions to effectively address their specific vulnerabilities. In this study, an inverse correlation of moderate magnitude achieved statistical significance between the tally of post-traumatic symptomatology, as ascertained by the Trauma Symptom Questionnaire (TSQ), and the likelihood of a probable diagnosis of PG, denoted by scores on the Brief Biosocial Gambling Screen (BBGS). This paradigmatic outcome stands in contrast to previous findings, which consistently posit an association between the breadth of experienced traumatic events across the lifespan—with an accentuated emphasis on the neglect encountered during early developmental stages (Horak et al., 2021, Hodgins et al., 2010)—and the subsequent manifestation of PG behaviors and the full clinical presentation of gambling disorders as documented in the work by Scherrer et al. (2007).

Both BPD traits and ASPD traits have been associated with PG earlier (Brown et al., 2015; Pietrzak & Petry, 2005: Blaszczynski and Nower, 2002, Blum et al., 2017, Martin et al., 2019, Pastwa-Wojciechowska, 2011). Traits indicative of BPD emerged as a salient predictor, for probable PG, while the propensity for drug misuse was significantly forecast by traits associated with antisocial personality disorders. Concurrently, problematic alcohol use was prognosticated by both sets of personality attributes. Clinically it is very important to notice that BDP is characterized by unstable interpersonal relationships, self-image disturbances, and pronounced impulsivity. The results of Brown et al. (2016) demonstrate that analogous biological and sociocultural determinants underlie the manifestation of both PG and personality disorders. Such factors encompass inadequate parent–child relationships during formative years, potential experiences of abuse, challenges in emotion regulation, substance misuse, and depressive and anxiety-related conditions. Individuals experiencing challenges with gambling, akin to those grappling with BPD, frequently exhibit traits of impulsivity, engage in interpersonal aggression, and tragically, are at heightened risk of suicide (APA, 2013).

While our research reveals that antisocial personality traits were not correlated with PG, other studies have found the opposite (Pietrzak & Petry, 2005; Blaszczynski and Nower, 2002, Blum et al., 2017, Martin et al., 2019, Pastwa-Wojciechowska, 2011). Previous discussions have highlighted an interesting point: even though some argue that antisocial personality disorder is not related to gambling-related crimes, other research suggests that traits of this disorder, like impulsiveness, could play a role in leading to these kinds of criminal acts. (Pastwa-Wojciechowska, 2011; May-Chahal et al., 2017). Our research did not show a direct link between PG and ASPD traits. The explanation for this could be that, based on the Pathways model (Blaszczynski & Nower, 2002), path three antisocial and impulsive gamblers may have characteristics of impulsivity and substance use disorders and a predisposition towards criminal behavior that are traits of both ASPD and BPD. Other explanation could be that there is an underlying comorbidity with BPD and ASPD, since ASPD is highly common in prison population. The co-occuring prevalence of BPD and ASPD is high among offenders, with prevalence rates ranging from 10.5. to 90,0 % (Armenti et al., 2017, Wetterborg et al., 2015, Robitaille et al., 2017). In addition, Anderson and colleagues (2021) highlighted that diagnostic clarity (BPD and ASPD) may be particularly difficult in forensic settings because there could be overlap and a lack of differentiation between PD constructs.

However, it is still important to consider the idea from Gorsane et al., (2017) that using substances along with PG could lower self-control and maybe result in criminal behavior. It is essential to note a study conducted by Helle et al. (2019), which emphasizes the significance of incorporating screening protocols, devising comprehensive treatment strategies, and implementing relapse prevention measures in addressing the intricate interplay of concurrent conditions, such as alcohol use disorder (AUD) and ASPD or BPD. Patients with PD traits, especially ASPD and BPD, may benefit Dialectical Behaviour Therapy DBT (Linehan 1993). DBT is a structured form of cognitive-behavioral therapy designed to treat complex emotional and behavioral disorders, particularly BPD and ASPD. It integrates principles of acceptance and change through a comprehensive treatment program. The DBT's structured approach, comprehensive skill training, and focus on both acceptance and change make it particularly effective for treating the complex behaviors and emotional regulation issues central to BPD and ASPD (Linehan, 1993, Fennessy, 2017).

The integration of personality disorder screening into standard rehabilitation or treatment protocols, particularly for incarcerated individuals, is imperative and warrants consideration. Given that individuals with both PG and personality disorders have a threefold increase in dropout rates compared to those with PG alone (Brown et al., 2016), implementing screening could assist practitioners to adopt a more empathetic stance toward non-adherence and to foster treatment compliance.

Our results underscore the importance of enhancing awareness among professionals who engage with individuals within the criminal justice sphere as an initial step toward enhancing the well-being of incarcerated individuals and mitigating the risk of recidivism (Castrén et al., 2021). Additionally, developing strategies to establish a continuum of support post-sentence is paramount, as is implementing appropriate follow-up measures to mitigate relapses and assess the efficacy of support systems within this specific population. Individuals who are incarcerated represent a marginalized demographic with evident requirements for social and healthcare provisions. Prior studies have acknowledged a spectrum of challenges, including physical health ailments, mental health concerns, diverse addictive tendencies, and social disparities (Banks et al., 2020; Sharman et al., 2019; Rudd & Thomas, 2016; Lind, 2022), all of which serve as significant hurdles to the well-being of prisoners. Individuals within carceral settings constitute an underserved population with discernible requirements for social and healthcare provisions, with previous research indicating their motivation to actively pursue assistance and support (Lind et al., 2019).

Treatments particularly for gambling disorder/PG in prison settings are still scarce. However, Jindani et al. (2021) investigated the PG treatment status in Canadian prisons and found that constraints related to availability and accessibility emerged as impediments to treatment, alongside factors such as inadequate awareness, structural hindrances (e.g., prison security protocols), stigma, resistance, and apprehension regarding potential adverse repercussions. In addition, gender specific needs were found that need to be addressed and studied further in the future. The study revealed a lack of consensus among the interviewed experts regarding the potential integration of PG treatment within substance use treatment programs.

Despite the well-documented prevalence of PG among incarcerated individuals, the insufficiency of screening measures and treatment initiatives for PG within criminal justice contexts persists globally. It is important to extend PG treatment services within the criminal justice system to ensure equitable access to healthcare and social welfare services for all. Moreover, such an approach is cost-efficient, given that undetected and untreated PG elevates the risk of recidivism. Determining whether incarcerated individuals with PG form a distinct subgroup within the prison population and among those with substance use disorders and personality disorders carries significant implications for shaping PG treatment protocols. Incarcerated individuals’ psychosocial well-being and social circumstances impact their recovery capital, encompassing physical, social, and cultural resources that individuals can leverage during their rehabilitation. These resources may include elements such as social support, coping skills, community engagement, and spirituality. Research has shown that higher recovery capital and spirituality predict greater progression in PG treatment. However, situational factors, such as stressful life events, may impede the positive impacts of recovery capital, with gender likely playing a role: among men, stressors such as interpersonal conflicts and financial strain have been found to negatively influence PG recovery (Gavriel-Fried et al., 2022; Gavriel-Fried, 2018, Gavriel-Fried et al., 2019a, Gavriel-Fried et al., 2019b).

## 6 Limitations

Our study had a small number of individuals with PG. Due to the small number of women with PG, we could not perform gender comparisons. Additionally, the assessment of PG and SUDs employed different timeframes (the past 12 months versus lifetime) and measurement tools (a short screening instrument versus a clinical interview), requiring careful consideration when interpreting the results. Furthermore, the study did not investigate whether the participants' sentences were directly linked to their gambling behavior. It is important to note that the mental health interview does not diagnose a psychiatric disorder, but instead reflects its preconditions.

## 7 Conclusions

The association between PG and criminal behaviors highlights the vulnerability of incarcerated individuals across diverse domains. It is crucial to conduct detailed assessments encompassing the nature of criminal activity, as well as the presence of personality disorders, especially BPD traits, and other comorbid conditions, to tailor rehabilitation and treatment strategies, with the aim of preventing recidivism. These findings hold substantial utility for clinicians engaged in the evaluation and management of addiction and mental health issues.

## 8 Disclaimer

Neither the Ministry of Social Affairs and Health, Helsinki, Finland, nor the Prison and Probation Service of Finland had any role in the study design, analysis, or interpretation of the results, nor in any phase of the publication process.

## 9 Ethics approval and consent to participate

The study was conducted in accordance with the ethical standards of the Declaration of Helsinki. The Ethics Committee of the Finnish Institute for Health and Welfare, Finland, approved the research protocol.

## Funding

The daily work of SC and JP was funded by the Ministry of Social Affairs and Health, Finland, within the objectives of Section 52 Appropriation of the Lotteries Act. However, it had no role in the study design, analysis, or interpretation of the results of the manuscript or any phase of the publication process. The Finnish Institute for Health and Welfare received funding from the Prison and Probation Service of Finland for a research project titled 'Problem Gambling Among Criminal Sanction Clients: Preventing Recidivism and Developing Support'. This funding included a salary for KL.

Conflicts of Interest: Authors confirm that there are no conflicts of interest, whether financial or non-financial, that could be perceived as relevant to the content of this manuscript.

Authors certify that the above statements are true and accurate to the best of my knowledge. Should any conflicts of interest arise during the review process or after publication, authors pledge to promptly disclose them to the Editor-in-Chief of the journal.

## CRediT authorship contribution statement

**Kalle Lind:** Writing – review & editing, Writing – original draft, Funding acquisition, Conceptualization. **Jussi Palomäki:** Writing – review & editing, Visualization, Formal analysis, Conceptualization. **Sari Castrén:** Writing – review & editing, Project administration, Funding acquisition, Conceptualization.

## Declaration of competing interest

The authors declare the following financial interests/personal relationships which may be considered as potential competing interests: The daily work of SC and JP was funded by the Ministry of Social Affairs and Health, Finland, within the objectives of Section 52 Appropriation of the Lotteries Act. However, it had no role in the study design, analysis, or interpretation of the results of the manuscript or any phase of the publication process. The Finnish Institute for Health and Welfare received funding from the Prison and Probation Service of Finland for a research project titled 'Problem Gambling Among Criminal Sanction Clients: Preventing Recidivism and Developing Support'. This funding included a salary for KL. Author SC works a part time private practitioner clinical psychologist at Addiktum Clinic Helsinki, Finland, treating mainly individuals with addiction problems, and at Mehiläinen Medical Center, Forum Helsinki, where she offers treatments to various psychological issues. She is a clinical advisor to the Canadian company Alavida, Vancouver (remote/internet treatment for alcohol disorder). She also trains professionals to treat gambling disorder with evidence-based methods as a part of her duty at the Finnish Institute for Health and Welfare, and addictions in general privately. She has received fees from Helsinki University, Tampere City, Vocational school Stadi, Lundbeck, the Finnish Association of Addiction Medicine, the Finnish Association on Intellectual and Developmental Disabilities (FAIDD), and Mehiläinen for her lectures on behavioural addictions and for training professionals, and writer's fees from the Finnish Medical Society Duodecim, Finnish Medical Journal and Myllyhoitoyhdistys ry. She received fees from Svenska Spel (Sweden) for evaluating grant proposals, and Tampere University for preliminary examination of PhD work. She declares no conflict of interest in relation to this manuscript. *Financial Interests*: Authors have no financial interests or affiliations with any organization or entity that could potentially influence or bias the content of this manuscript. *Non-Financial Interests*: Authors declare no non-financial interests, including personal relationships, political or religious beliefs, or any other factors that may influence my objectivity or impartiality in the publication of this manuscript.

## Data Availability

The data that has been used is confidential.
