# Supplementary material for: Examining problem gambling, substance use disorders and cluster B personality traits among incarcerated individuals
Source: Addict Behav Rep. 2024 Oct 9;20:100566. doi: 10.1016/j.abrep.2024.100566 (PMC11531610; doi:10.1016/j.abrep.2024.100566)
Supplement: Supplementary Data 1 [file mmc1.docx]

**APPENDIX 1. Requested variables.**

| Variables grouped by category | Variable names |
| --- | --- |
| Background information |  |
| Gender | gender |
| Age | age |
| Marital status | marital_status |
| Housing type | housing |
| Childhood living situation | housing_childhood |
| Parents' divorce | parents_divorced |
| Basic education | education |
| Education | vocational_training |
| Employment status before incarceration | employment |
| Social situation |  |
| Future support | after_sentence, after_sentence_conditions |
| Basic needs and social network | support.support_conditions, social_contacts.social_contacts_type,  social_life.social_life_feelings |
| Religiosity, spirituality | religion, sprituality |
| Trauma and abuse |  |
| Trauma | tsq.tsq_feature |
| Physical abuse | physical_abuse |
| Sexual abuse | sexual_abuse |
| Psychological abuse | mental_abuse |
| Economic abuse | economic_abuse.set, economic_abuse_other |
| Addictions |  |
| Gambling (BBGS, 3 questions) | bbgs_posit, gambling, gambling_hide, gambling_problems |
| Drug use (scale) | drugs_iv, drugs_shared_needle, drugs_shared_equipment, drugs_needle_reuse, dast, polydrug_use.polydrug_use_type |
| Alcohol dependence (AUDIT and SADD, before incarceration) | drinking_frequency, drinking_amount, drinking_habits, drinking_reckless, drinking_consequences, drinking_morningafter, drinking_guilt, drinking_memoryloss, drinking_accident, drinking_worried |
| Smoking | smoking, smoking_amount |
| Mental health and health |  |
| Mood, psychological distress, resilience | deps, cd_risc.cd_risc_scale, depression, anxiety |
| Loneliness | loneliness |
| Somatic health | health, longterm_illness, 15d |
| Disturbance and autism | adhd.adhd_feature,  austism_spectrum.autism_spectrum_feature:1-10 |
| Alexithymia | alexithymia.alexithymia_scale |
| Involvement and meaningfulness |  |
| Social support and involvement | sokra.sokra |
| Life satisfaction | life_satisfaction |
| Diagnostic summaries | SCID 1-2, DIVA |
